# Evidence of human leptospirosis cases in a cohort of febrile patients in Bangui, Central African Republic: a retrospective study, 2012–2015

**DOI:** 10.1186/s12879-018-3298-z

**Published:** 2018-08-07

**Authors:** Pierre-Alain Rubbo, Marie-Estelle Soupé-Gilbert, Davy Martial Golongba, Florent Mbombo, Dominique Girault, Emmanuel Nakouné, Jean-Pierre Lombart, Sébastien Breurec, Cyrille Goarant

**Affiliations:** 1 Institut Pasteur, BP 923 Avenue de l’Indépendance, Bangui, Central African Republic; 2 Institut Pasteur, Noumea, New Caledonia; 3 University Hospital of Pointe-à-Pitre/Abymes and University of Antilles, Pointe-à-Pitre, Guadeloupe France

**Keywords:** Leptospirosis, Bacteria, Infection, Central African Republic, Sub-Saharan Africa, Surveillance, Emergence

## Abstract

**Background:**

In spite of a local favorable environment, leptospirosis has never been described in Central African Republic so far mainly because of the weakness of diagnostic tests and differential diagnostic strategy for febrile jaundice cases negative for yellow fever virus. Here we bring a complementary insight to conclusions of Gadia CLB et al. regarding the presence of leptospirosis in Central African Republic in YFV-negative febrile icteric patients.

**Methods:**

Our study included 497 individuals presenting with fever and jaundice but negative for yellow fever infection, retrospectively selected from the national surveillance biobank for yellow fever in Institut Pasteur de Bangui, Central African Republic.

A combination of serological (ELISA, agglutination) and molecular biology techniques (quantitative real-time polymerase chain reaction) was used to identify *Leptospira* or the patient’s immune response to the bacteria. Statistical analyses were done using the non parametric Mann-Withney U test with a 5% statistical threshold.

**Results:**

ELISA test results showed 46 positive serum samples while 445 were negative and 6 remains equivocal. In addition, the reference microscopic agglutination test for leptospirosis diagnostic confirmed that 7 out of 32 samples tested were positive. Unfortunately, all 497 serum samples tested for leptospirosis were negative using the molecular techniques.

**Conclusions:**

Unlike Gadia et al., we confirmed that leptospirosis is circulating in Central African Republic and therefore may be responsible for some of the unexplained cases of febrile jaundice in the country. Thus, leptospirosis needs to be investigated to improve identification of aetiological pathogens. Our study also suggests a need to improve sample transportation and storage conditions.

## Background

Cases of human leptospirosis have never been described in Central African Republic despite local suitable environmental conditions for disease dissemination and human infection. In addition, among cases suspected of yellow fever, a large majority are negative for Yellow Fever Virus (YFV) highlighting the need to improve differential diagnostic and identify other pathogens responsible for febrile jaundice, including *Leptospira.* Gadia CLB et al. have recently explored pathogens that could be responsible for febrile jaundice in individuals negative for YFV infection in Central African Republic (CAR).

Their retrospective study highlighted that almost half of the 198 samples tested were positive for at least one pathogen other than YFV [1]. This informative article showed that hepatitis viruses are predominant in YFV-negative Yellow Fever suspicions. It confirmed the importance of establishing a differential diagnostic algorithm for patients with febrile jaundice, and of improving the management of such cases by healthcare workers, especially in resources-limited countries, such as in sub-saharan countries where national surveillance of malaria and YFV do not include differential testing of related pathogens. Finally, their study also highlighted the scarcity of reliable data from African countries about the role of of pathogens other than YFV and *Plasmodium falciparum* in febrile jaundice cases.

CAR is one of the poorest countries in the world facing a chronic healthcare crisis due to successive political conflicts in the past years. Infections frequently associated to febrile jaundice, such as YFV, malaria, hepatitis or leptospirosis for example, are major public health issues in this country. More importantly, although very few YFV-infected individuals are confirmed each year in the country [2] along the World Health Organization (WHO) epidemiological surveillance program, no public health strategies have been implemented for differential diagnostic of febrile jaundice. Therefore it remains crucial to better evaluate the impact and prevalence of each pathogen associated with febrile jaundice in CAR with the aim to improve therapeutic management of such patients. Here we bring a complementary insight regarding the presence of leptospirosis in Central African Republic in individuals uninfected by YFV.

## Methods

We designed a retrospective study using 497 YFV-negative serum samples from individuals with febrile jaundice, and stored in the YFV surveillance program biobank between 2012 and 2015 in Institut Pasteur de Bangui.

All 497 individual samples were tested with the Panbio Leptospira Immunoglobulin M (IgM) Enzyme-linked immunosorbent assay (ELISA) kit (Alere, Jouy-en-Josas, France) according to manufacturer’s instructions.

In addition, a panel of 32 serum samples selected from those previously tested in ELISA were also tested using the reference microscopic agglutination test (MAT) for leptospirosis diagnostic [3] using a 24-serovar panel based on the WHO recommendation [4]. This panel included serovars Australis, Autumnalis, Ballum, Bataviae, Canicola, Panama, Pomona, Pyrogenes, Tarassovi, Celledoni, Cynopteri, Djasiman, Gryppotyphosa, Hebdomadis, Javanica, Louisiana, Mini, Sejroe, Shermani (all from eponymous serogroups) as well as Icterohaemorrhagiae and Copenhageni (serogroup Icterohaemorrhagiae), Hardjo (serogroup Sejroe) and Bratislava (serogroup Australis) and the saprophytic strain Patoc (serogroup Semaranga).

All 497 serum samples were also tested for leptospirosis using the LipL32-based quantitative Polymerase Chain Reaction (qPCR) [5]. A subset was also tested with the RNAseP qPCR as an external control PCR [5] as well as with additional molecular detection assays targeting lipL32 [6] or the 16S rRNA gene [7].

Statistical analyses were done using the non parametric Mann-Withney U test with a 5% significance threshold.

## Results

A majority of individuals selected in this study were below 19 years old (62.8%) while a minority of individuals were above 30 years old (18.1%) (Table 1). Only 7 out of the 497 (1.4%) individual samples were sampled within the day of symptoms onset, and only 130 out of the 497 (26.2%) individual samples were received at the laboratory and stored within the same day of serum sampling (Table 1).

**Table 1:** Characteristics of individuals with febrile jaundice but negative when tested for YFV according to results of ELISA IgM tests for leptospirosis

Characteritics		Positive Leptospirosis IgM ELISA test		Negative Leptospirosis IgM ELISA test		Equivocal Leptospirosis IgM ELISA test		*P* value^*^
		No	%	No	%	No	%	
Age (years)	0–9	6	14.3	190	43.8	3	14.3	0.07
	10–19	13	31.0	92	21.2	8	38.1	0.86
	20–29	13	31.0	77	17.7	5	23.8	0.17
	30–39	4	9.5	35	8.1	2	9.5	0.35
	40–49	2	4.7	27	6.2	2	9.5	0.76
	> 50	4	9.5	13	3.0	1	4.8	0.36
Time interval between symptoms and serum sampling (days)	< 1	0	0.0	6	1.4	1	4.8	NA
	1–4	14	33.3	180	41.5	7	33.3	0.09
	5–10	28	66.6	247	56.9	13	61.9	0.26
	> 10	0	0.0	1	0.2	0	0.0	NA
Time interval between serum sampling and lab storage (days)	< 1	7	16.7	120	27.6	3	14.3	NA
	1–4	25	59.5	227	52.3	13	61.9	0.53
	5–10	9	21.4	71	16.4	3	14.3	0.34
	> 10	1	2.4	16	3.7	2	9.5	0.35
	Total	42	100	434	100	21	100	

^*^Comparison was done using the nonparametric Mann-Whitney *U* test between negative and positive Leptospirosis IgM ELISA tests. Statistical significance was assessed at *P* value < 0.05

Among them, 42 were positive in ELISA with a mean absorbance of 1.76 (range 1.2–4.5), 434 were negative with a mean absorbance of 0.31 (range 0.1–0.8), and 21 were equivocal (i.e. with a value between 0.9 and 1.1) with a mean of 0.97 (range 0.9–1.1). Globally, median age of individuals included in the study was 14 years old (range 0–76). Median age of individuals with a positive ELISA test was 21.5 years, while it was 12 years old for individuals with a negative ELISA test. All the samples with an equivocal ELISA results were retested with the same ELISA kit: 11 samples became negative, 4 became positive and 6 remained equivocal.

Among the 32 serum samples tested in MAT, 9 were positive with a titer ≥50 including 7 with a titer ≥100, suggesting the presence of specific antibodies against *Leptospira*. All 7 samples with a titer ≥100 in MAT were also positive in ELISA confirming an earlier infection in these individuals. Two samples had titer of 800 (Patoc) and 1600 (Bratislava) suggesting a recent infection and demonstrating a circulation of the disease in the country. The titer of 800 for the non-pathogenic serovar Patoc suggests that the MAT panel used did not include an appropriate representative of the infecting serovar.

Regarding molecular detection of leptospirosis, all 497 serum samples were negative with the LipL32 qPCR. The RNAseP qPCR gave positive amplification, however with late cycle thresholds (Ct), suggesting that the DNA extracts only contained low amounts of DNA.

## Discussion

Suboptimal sampling, transportation (from remote field sites and between laboratories) and storage conditions may have led to the degradation of *Leptospira* DNA, leading to a very low sensitivity. Other molecular tests also failed to evidence *Leptospira* DNA in the specimens. Thus, efforts must be made to optimize pre-analytical management of samples, notably the time between sampling on the field and diagnostic test in the laboratory to improve identification of pathogens by limiting the risk of sample alteration, which may lead to false negative molecular tests for leptospirosis and other pathogens.

Although we failed to detect *Leptospira* DNA by qPCR possibly because of the degradation of bacterial DNA due to time between sampling and testing, serological tests confirmed that leptospirosis is circulating in Central African Republic. This is the first study revealing proofs of circulation of *Leptospira* in Central African Republic. Globally, very few information is available on leptospirosis incidence, distribution and burden in sub-saharan countries [8].

In addition to hepatitis viruses as demonstrated by Gadia et al. [1], *Leptospira* may also be responsible for some of the unexplained cases of febrile jaundice disease identified in the country and would deserve to be investigated within a differential diagnostic strategy, for example within the national surveillance program of YFV.

## Conclusions

This study showed that, despite leptospirosis is rarely mentioned, strains of *Leptospira* circulate in Central African Republic. Leptospirosis diagnostic should be included in the toolkit of laboratories when febrile jaundice disease is suspected. If left untreated, the disease may cause organ failure leading to death. Rigorous procedures for pre-analytical management of samples may also improve the capacity of laboratories to identify pathogens such as *Leptospira.*

## Abbreviations

CAR: Central African Republic
DNA: DesoxyriboNucleic Acid
ELISA: Enzyme-linked immunosorbent assay
IgM: Immunoglobulin M
MAT: microscopic agglutination test
PCR: polymerase chain reaction
qPCR: quantitative polymerase chain reaction
WHO: World Health Organization
YFV: Yellow Fever virus
